# Association between sleep insufficiency and dyslipidemia: a cross-sectional study among Greek adults in the primary care setting

**DOI:** 10.5935/1984-0063.20200124

**Published:** 2022

**Authors:** Dimitrios Tsiptsios, Eleni Leontidou, Petros N. Fountoulakis, Andreas Ouranidis, Anestis Matziridis, Apostolos Manolis, Andreas S. Triantafyllis, Konstantinos Tsamakis, Aspasia Serdari, Aikaterini Terzoudi, Elena Dragioti, Paschalis Steiropoulos, Gregory Tripsianis

**Affiliations:** 1 South Tyneside & Sunderland NHS Foundation Trust, Department of Clinical Neurophysiology - Sunderland - Tyne & Wear - United Kingdom.; 2 Democritus University of Thrace, Laboratory of Medical Statistics - Alexandroupolis - Thrace - Greece.; 3 Askepeion Hospital, Department of Cardiology - Athens - Attiki - Greece.; 4 Aristotle University of Thessaloniki, Department of Pharmaceutics - Thessaloniki - Central Macedonia - Greece.; 5 King’s College, Institute of Psychiatry, Psychology and Neuroscience - London - London - United Kingdom.; 6 Democritus University of Thrace, Department of Child and Adolescent Psychiatry - Alexandroupolis - Thrace - Greece.; 7 Democritus University of Thrace, Neurology Department - Alexandroupolis - Thrace - Greece.; 8 Linköping University, Department of Health, Medicine and Caring Sciences - Linköping - Linköping - Sweden.; 9 Democritus University of Thrace, Department of Pneumonology - Alexandroupolis - Thrace - Greece.

**Keywords:** Cross-Sectional Study, Sleep Duration, Dyslipidemia, Sleep Quality, Insomnia

## Abstract

**Objective:**

To investigate the potential association between sleep insufficiency and dyslipidemia (DL) in the primary care setting using self-reported questionnaires.

**Material and Methods:**

957 adults aged between 19 and 86 years old from the rural area of Thrace, Greece were enrolled in this cross-sectional study. Multistage stratifed cluster sampling was used and the subjects were classifed into three groups according to sleep duration [short (<6h), normal (6-8h), and long (>8h) sleep duration]. DL was defined by a positive response to the question “Have you ever been told by a doctor or health professional that your blood cholesterol or triglyceride levels were high?”, or if they were currently taking antilipidemic agents. Sleep quality, utilizing Epworth sleepiness scale, Athens insomnia scale, Pittsburgh sleep quality index and Berlin questionnaire, was also examined.

**Results:**

DL prevalence was significantly associated with short sleep duration (aOR=2.18, p<0.001) and insomnia (aOR=1.43, p=0.050), while its relation with poor sleep quality (aOR=1.31, p=0.094) and risk for obstructive sleep apnea (aOR=1.32, p=0.097) were of marginal statistical significance. Concerning insomnia subtypes, DL was significantly associated with difficulties maintaining sleep (aOR=2.99, p<0.001) and early morning awakenings (aOR=1.38, p=0.050), but not difficulties initiating sleep (aOR=1.18, p=0.328).

**Conclusion:**

This study reveals an association between sleep pathology and DL. Thus, early pharmacological and cognitive or behavioral interventions that improve sleep are deemed necessary in order to decrease DL burden.

## INTRODUCTION

Cardiovascular disease (CVD) comprises a major public health issue, as it constitutes the leading cause of non-communicable disease mortality worldwide^1^. CVD pathogenesis is complex including non-modifable risk factors, such as age, gender, race and ethnicity, and modifable risk factors, such as smoking, lack of physical activity, diabetes mellitus, hypertension, and visceral obesity^2^. Dyslipidemia (DL) defined as high levels of total cholesterol (TC), low-density lipoprotein cholesterol (LDL-C), triglycerides (TG), and low levels of high-density lipoprotein cholesterol (HDL-C), is another well-established reversible risk factor for CVD, thus encompassing an important issue in the field of health promotion and CVD prevention^3,4^.

Serum lipid and lipoprotein levels are infuenced by lifestyle parameters, such as smoking, alcohol consumption, and exercise^5,6^. Due to public health concerns about the declining quality and quantity of sleep in modern society, research into the effect of sleep on health has blossomed. Although in recent years, both short and long sleep duration have been associated with chronic diseases such as hypertension^7^, diabetes mellitus^8^, and CVD^9^, results on the potential relationship between sleep duration and DL have been contradictory so far, as U-shaped associations^10,11^ or relationships between DL and only short^12^ or long sleep duration^13^ have been presented. Apart from that, few studies have investigated the effect of sleep quality on DL reaching antithetical conclusions, as well^14,15,16^. Furthermore, there exists no consensus on the probable gender-specific association between sleep pathology and DL^17,18^. Finally, few European studies have investigated the potential association between sleep pathology and DL^12,19^.

Our research group utilizing self-reported questionnaires has recently exhibited that sleep pathology is associated with increased prevalence of anxiety^20^, depression^21^, diabetes mellitus^22^, and hypertension^23^. In this paper, we aimed to investigate possible correlations between sleep quantity/quality and DL considering several socio-demographic characteristics, lifestyle habits and health related characteristics of the participants and focusing on potential gender-specific associations.

## MATERIAL AND METHODS

### Study sample, research design and covariates

The study population in this cross-sectional study consisted of 957 participants, 439 (45.9%) males and 518 (54.1%) females, with a mean age of 49.62±14.79 years (range, 19-86 years; median age, 50 years). The research design of this study is reported in Serdari et al. (2020)^20^. The questionnaires used in order to collect standard socio-demographic characteristics, lifestyle, and dietary habits and health related characteristics of the participants are reported in Matziridis et al. (2020)^22^. Chronic disease morbidity was defined as the self-reported preexisting (for ≥3 months over the past year) health problems, such as: hypertension, diabetes mellitus, cancer, cardiovascular, rheumatic, gastrointestinal, pulmonary, or neurologic disease.

## ETHICS

All procedures performed in the study were in accordance with the ethical standards of the Democritus University Ethics Committee, which approved its conduct, and with the standards of the Helsinki declaration (1964) and its later amendments. Informed consent was obtained from all participants of the study.

### Estimation of sleep duration and sleep efficiency

Participants answered the following questions: “At what time do you normally go to bed?”, “At what time do you normally get up?”, and “On average, how many hours do you sleep every night?” for an average weekday and weekend day over the previous month. Time in bed, sleep duration, and sleep efficiency calculation formulae on weekdays, weekend days and weighted mean measures utilized in this study are reported in Matziridis et al. (2020)^22^. According to calculated sleep duration, participants were then classifed into: short sleepers (<6 hours), normal sleepers (6-8 hours), and long sleepers (>8 hours)^24,25^.

### Evaluation of sleep quality

Sleep quality was assessed with the Greek versions of Epworth sleepiness scale (ESS)^26^, Athens insomnia scale (AIS)^27^, Pittsburgh sleep quality index (PSQI)^28^, and Berlin Questionnaire (BQ)^29^ that evaluate excessive daytime sleepiness, insomnia, sleep quality, and risk of obstructive sleep apnea (OSA), respectively. With regards to insomnia characteristics, participants were asked whether they experienced difficulties initiating or maintaining sleep or early morning awakenings.

### Definition of DL

DL was defined by a positive response to the question “Have you ever been told by a doctor or health professional that your blood cholesterol or triglyceride levels were high?” or if they were currently taking antilipidemic agents^30,31^.

### Statistical analysis

Statistical analysis of the data was performed using IBM Statistical Package for the Social Sciences (SPSS), version 19.0 (IBM Corp., Armonk, NY, U.S.A.). The normality of quantitative variables was tested with Kolmogorov-Smirnov test. Quantitative variables were expressed as mean±standard deviation (SD) and qualitative variables were expressed as absolute and relative (%) frequencies. In particular, mean estimated time of sleep characteristics (i.e., bedtime, rise time, time in bed, and sleep duration) were expressed as HH:MM. We conducted the following analyses: (i) in the univariate analysis, the association of DL with subjects’ characteristics, sleep characteristics, and sleep disorders were assessed using the chi-square test and Student’s t-test; (ii) multivariate stepwise logistic regression analysis was used to explore the independent risk factors for DL, controlling for all subjects’ characteristics; (iii) for the evaluation of the effect of sleep duration and sleep disorders on the prevalence of DL, two different logistic regression models were constructed: model 1 (crude, unadjusted) and model 2 (adjusted for subjects’ socio-demographic characteristics: gender, age, marital status, cultural status, place of residence, education level, working status, financial status; lifestyle habits: smoking status, alcohol consumption, daily coffee consumption, caffeine consumption in the evening, adherence to the Mediterranean diet, time watching TV or using a computer before bedtime, physical activity, nap during the day; and health related characteristics: subjective general health status, BMI, chronic disease morbidity, anxiety, depression, and use of sleep medication). Odds ratios (OR) with their 95% confidence intervals (CI) were estimated as the measure of the above associations.

Receiver operating characteristic (ROC) analysis was used to provide the ability of sleep duration to classify subjects with DL. The area under the ROC curve (AUC), sensitivity, and specificity were estimated. The optimal cutoff value of the sleep duration that differentiates DL from non-DL individuals was derived according to Youden index. All tests were two tailed and statistical significance was considered for *p*-values≤0.05.

## RESULTS

### Subjects’ characteristics

Participants’ socio-demographic, lifestyle and health related characteristics are summarized in Tables 1 and 2. The average self-reported sleep duration was 6hrs and 19min on the weekdays and 6hrs and 45min on the weekends; 31.7% and 22.9% of the sample reported sleep duration less than 6hrs, while 7.9% and 14.2% of the sample reported sleep duration more than 8hrs per night on the weekdays and on the weekends, respectively. Sixty-six participants (6.9%) were on sleep related medications.

**Table 1.** Prevalence of dyslipidemia in relation to subjects’ demographic characteristics.

	Dyslipidemia			
	Total sample	Frequency	Proportion (%)	*p*-value
**Gender**				0.310
Females	518 (54.1)	116	31.1	
Males	439 (45.9)	150	34.2	
**Age (years)**				<0.001
≤30	132 (13.8)	9	6.8	
30 - 40	141 (14.7)	24	17.0	
41 - 50	232 (24.2)	51	22.0	
51 - 60	212 (22.2)	71	33.5	
61 - 70	145 (15.2)	81	55.9	
>70	95 (9.9)	75	78.9	
**Marital status**				<0.001
Married	645 (67.4)	220	34.1	
Single	196 (20.5)	23	11.7	
Divorced	36 (3.8)	8	22.2	
Widowed	80 (8.4)	60	75.0	
**Cultural status**				0.020
Greek Christians	632 (66.1)	189	29.9	
Greek Muslims	273 (28.5)	107	39.2	
Expatriated Greeks	52 (5.4)	15	28.8	
**Place of residence**				<0.001
Urban	416 (43.5)	84	20.2	
Rural	541 (56.5)	227	42.0	
**Education level**				<0.001
Low	313 (32.7)	158	50.5	
Medium	340 (35.5)	98	28.8	
High	304 (31.8)	55	18.1	
**Working Status**				0.927
Employed	872 (91.1)	283	32.5	
Unemployed	85 (8.9)	28	32.9	
**Financial status (n=812)**				<0.001
Low	476 (49.7)	183	38.4	
Medium	200 (20.9)	68	34.0	
High	136 (14.2)	20	14.7	

**Table 2.** Prevalence of dyslipidemia in relation to subjects’ lifestyle habits and health related characteristics.

	Dyslipidemia			
	Total sample	Frequency	Proportion (%)	p-value
Smoking ever				0.001
Never smoking	369 (38.6)	136	36.9	
Ex-smoker	255 (26.6)	93	36.5	
Current smoking	333 (34.8)	82	24.6	
Alcohol consumption				0.005
Never	488 (51.0)	179	36.7	
Occasionally or daily	469 (49.0)	132	28.1	
Coffee consumption				0.145
None	84 (8.8)	18	21.4	
1 - 2 cups/day	564 (58.9)	189	33.5	
3 - 4 cups/day	260 (27.2)	89	34.2	
>4 cups/day	49 (5.1)	15	30.6	
Caffeine consumption in the evening (>6 p.m.)				<0.001
No	415 (43.4)	160	38.6	
Yes	542 (56.6)	151	27.9	
Adherence to MED diet				0.004
Low	743 (77.6)	259	34.9	
High	214 (22.4)	52	24.3	
Time watching TV or using a computer before bedtime <1 hour	120 (12.5)	25	20.8	<0.001
1 - 2 hours	326 (34.1)	90	27.6	
>2 hours	511 (53.4)	196	38.4	
Physical activity				<0.001
Low	805 (84.1)	293	36.4	
High	152 (15.9)	18	11.8	
Nap during the day				0.834
No	721 (75.3)	233	32.3	
Yes	236 (24.7)	78	33.1	
Subjective health status				<0.001
Bad	220 (23.0)	135	61.4	
Good	737 (77.0)	176	23.9	
BMI status				<0.001
Normal	328 (34.3)	66	20.1	
Overweight	272 (28.4)	82	30.1	
Obese	357 (37.3)	163	45.7	
Chronic disease morbidity				<0.001
No	585 (61.1)	84	14.4	
Yes	372 (38.9)	227	61.0	
Anxiety symptoms				lt;0.001
No	635 (66.4)	166	26.1	
Yes	322 (33.6)	145	45.0	
Depression symptoms				<0.001
No	685 (71.6)	180	26.3	
Yes	272 (28.4)	131	48.2	
Use of sleep medication				0.020
No	891 (93.1)	281	31.5	
Yes	66 (6.9)	30	45.5	

A total of 311 participants (32.5%) were classifed with DL. The prevalence of DL in relation to participants’ socio-demographic, lifestyle and health related characteristics is summarized in Tables 1 and 2. Significant determinants of DL obtained by multivariate logistic regression models are presented in Table 3.

**Table 3.** Significant determinants of dyslipidemia obtained by multivariate logistic regression models.

Characteristics	aOR	95% CI	*p*-value
Age (10-year increase)	1.84	1.59 – 2.13	<0.001
Widowed	2.74	1.44 – 5.20	0.002
Low education level	2.69	1.47 – 4.91	0.001
Low or medium financial status	1.91	1.10 – 3.31	0.022
Never or ex-smoking	1.67	1.12 – 2.49	0.011
No alcohol consumption	1.50	1.04 – 2.18	0.031
Low adherence to Mediterranean diet	1.67	1.09 – 2.57	0.019
Obesity	2.58	1.79 – 3.70	<0.001
Anxiety symptoms	2.71	1.93 – 3.81	<0.001

Notes: aOR = Adjusted odds ratio; CI = Confidence interval; all subjects’ socio-demographic, lifestyle habits and health related characteristics were included in the model; All variables (with the exception of age) were binary (no, yes); category "no" forms the reference group.

### DL and sleep habits

The association of DL with subjects’ sleep characteristics is shown in Table 4. The average weekly time in bed and sleep duration were calculated and compared between the two groups. It was noted that, although subjects with DL spent 11min longer time in bed (*p*=0.007), they reported a 22min shorter sleep duration (*p*<0.001) and lower sleep efficiency (*p*<0.001) compared to those without DL. All the above relations between DL and sleep characteristics remained unchanged among females and males. In particular, females with DL used to sleep 21min less than females without DL (*p*=0.005) and males with DL used to sleep 22min less than males without DL (*p*=0.003). Among subjects with DL, all three sleep characteristics were similar between males and females (time in bed: *p*=0.085; sleep duration: *p*=0.303; sleep efficiency: *p*=0.964).

**Table 4.** Association of dyslipidemia with sleep characteristics.

	Dyslipidemia				
	Total sample	No	Yes	Difference*	*p*-value
**Weekday sleep habits**					
Bedtime	11:29 (1:05)	11:41 (1:06)	11:03 (0:56)	-38 (2.6)	<0.001
Rise time	6:53 (1:01)	7:00 (1:02)	6:39 (0:55)	-21 (2.5)	<0.001
Time in bed	7:24 (1:05)	7:19 (1:04)	7:36 (1:07)	17 (2.6)	<0.001
Sleep duration	6:19 (1:11)	6:24 (1:06)	6:09 (1:20)	-15 (2.9)	0.004
Sleep efficiency (%)	86 (12)	88 (11)	81 (12)	-7 (0.8)	<0.001
**Weekend sleep habits**					
Bedtime	11:55 (1:19)	12:15 (1:17)	11:14 (1:04)	-61 (3.0)	<0.001
Rise time	7:46 (1:32)	8:05 (1:32)	7:05 (1:15)	-60 (3.6)	<0.001
Time in bed	7:50 (1:00)	7:50 (1:00)	7:51 (1:01)	1 (2.5)	0.702
Sleep duration	6:45 (1:16)	6:58 (1:09)	6:19 (1:21)	-39 (3.0)	<0.001
Sleep efficiency (%)	86 (12)	88 (10)	81 (12)	-7 (0.8)	<0.001
**Weekly sleep habits Total sample**					
Time in bed	7:32 (1:00)	7:28 (0:58)	7:39 (1:03)	11 (2.5)	0.007
Sleep duration	6:26 (1:10)	6:34 (1:04)	6:12 (1:19)	-22 (2.9)	<0.001
Sleep efficiency (%)	86 (12)	88 (10)	81 (12)	-7 (0.8)	<0.001
**Females**					
Time in bed	7:36 (0:59)	7:32 (1:00)	7:45 (0:56)	13 (3.4)	0.019
Sleep duration	6:30 (1:10)	6:37 (1:05)	6:16 (1:19)	-21 (4.0)	0.005
Sleep efficiency (%)	86 (12)	88 (11)	81 (13)	-7 (1.1)	<0.001
**Males**					
Time in bed	7:26 (1:00)	7:23 (0:54)	7:33 (1:08)	10 (3.6)	0.142
Sleep duration	6:22 (1:10)	6:29 (1:02)	6:07 (1:19)	-22 (4.0)	0.003
Sleep efficiency (%)	86 (11)	88 (10)	81 (10)	-7 (1.0)	<0.001

Notes: *mean difference (S.E.) between subjects with and without dyslipidemia is expressed as minutes (bedtime, rise time, time in bed, and sleep duration) and percentages (sleep efficiency).

In the sequence, according to the self-reported sleep duration, participants were categorized into three groups: short (<6h), normal (6-8h), and long (>8h) sleep duration. The association between the development of DL and sleep duration, which was considered as a categorical variable (Table 5) revealed that DL was significantly more frequent (*p*<0.001) in subjects with short (53.1%) compared to those with normal (25.8%) and long (30.9%) sleep duration. The association of DL with sleep duration status had the same pattern in both genders (*p*=0.002 for females; *p*<0.001 for males) (Table 5). In particular, logistic regression analysis revealed that in subjects with short sleep duration there were more than 3-times higher odds for DL compared to subjects with normal sleep duration (OR=3.26, *p*<0.001). A 2.65-fold (*p*<0.001) and a 3.92-fold (*p*<0.001) increase in odds of DL was associated with short sleep duration in women and men, respectively.

**Table 5.** Association of sleep duration with dyslipidemia (DL) using logistic regression models.

			Model 1		Model 2	
	DL, n (%)	p-value	cOR (95% CI)	p-value	aOR (95% CI)	p-value
**Total**						
Sleep duration		<0.001				
Short	111 (53.1)		3.26 (2.35-4.51)	<0.001	2.18 (1.50-3.19)	<0.001
Normal	158 (25.8)		Ref.		Ref.	
Long	42 (30.9)		1.28 (0.86-1.93)	0.228	1.02 (0.64-1.64)	0.932
**Females**						
Sleep duration		0.002				
Short	45 (48.9)		2.65 (1.65-4.25)	<0.001	2.01 (1.10-3.68)	0.023
Normal	94 (26.6)		Ref.		Ref.	
Long	22 (30.6)		1.22 (0.70-2.12)	0.487	1.34 (0.70-2.59)	0.384
**Males**						
Sleep duration		<0.001				
Short	66 (56.4)		3.92 (2.47-6.23)	<0.001	2.84 (1.69-4.75)	<0.001
Normal	64 (24.8)		Ref.		Ref.	
Long	20 (31.1)		1.38 (0.76-2.51)	0.295	0.91 (0.47-1.77)	0.787

Notes: cOR = Crude odds ratio; aOR = Adjusted odds ratio; CI = Confidence interval; Model 1 = Crude, unadjusted; Model 2 = Adjusted for socio-demographic characteristics, lifestyle habits (smoking status, alcohol consumption, daily coffee consumption, caffeine consumption in the evening, adherence to the Mediterranean diet, time watching TV or using a computer before bedtime, physical activity, nap during the day) and health related characteristics (subjective general health status, BMI, chronic disease morbidity, anxiety, depression, and use of sleep medication).

### Independent effect of DL on sleep habits

Two separate multivariate logistic regression models, controlling for the effect of all subjects’ socio-demographic, lifestyle, and health related characteristics, were constructed in order to assess the independent effect of sleep duration on the prevalence of DL. When sleep duration was entered in the model as a continuous variable, it remained a statistically significant independent determinant of DL (*p*=0.049); in particular, shorter sleep duration by one hour was associated with a 14%-increase in the odds for DL (aOR=1.14, 95% CI=1.00-1.30).

When sleep duration was entered in the multivariate logistic regression model as a categorical variable, the inverse relationship between DL and sleep duration persisted even after the adjustment for all subjects’ characteristics. In particular, the odds of DL were more than 2 times higher in subjects sleeping less than 6 hours (aOR=2.18, *p*<0.001) relative to those with normal sleep duration; the respective odds for DL were similar in the two genders (aOR=2.01, *p*=0.023 in females; aOR=2.84, *p*<0.001 in males). Sleeping longer than 8 hours showed no significant effect on the development of DL (Table 5).

Moreover, the area under the ROC curve (AUC) showed that sleep duration has a significant ability to discriminate subjects with DL (AUC=0.633, 95% CI=0.592-0.675, *p*<0.001). The optimal cutoff point of sleep duration of 5:33 hours, which was determined to classify subjects with DL, yielded high sensitivity of 50% and specificity of 85%. Sleep duration showed significant discrimination ability in both genders, although its performance was superior among males (females: AUC=0.578, 95% CI=0.520-0.637, *p*=0.005, cutoff ≤5:33 hours, sensitivity=39%, and specificity=85%; males: AUC=0.688, 95% CI=0.630-0.746, *p*=0.030, cutoff ≤5:38 hours, sensitivity=60%, and specificity=86%).

### DL and sleep disorders

According to the Greek versions of ESS, AIS, PSQI and BQ the prevalence of daytime sleepiness was 8.7% (83 subjects), insomnia 18.0% (172 subjects), poor sleep quality 38.5% (368 subjects), and high risk of obstructive sleep apnea 36.4% (348 subjects). The internal consistency of all four questionnaires was very high (Cronbach α coefficient was ranged from 0.74 to 0.88). The presence of DL in relation to sleep disorders is shown in Table 6.

**Table 6.** The association of sleep questionnaires and sleep difficulties with dyslipidemia (DL) using logistic regression models.

			Model 1		Model 2	
	DL	*p*-value	cOR (95% CI)	*p*-value	aOR (95% CI)	*p-*value
**Sleep questionnaires**						
ESS		0.466				
Normal day sleepiness	287 (32.8)		Ref.		Ref.	
Excessive day sleepiness	24 (28.9)		0.83 (0.51-1.37)	0.466	0.72 (0.44-1.20)	0.212
AIS		0.011				
Non-insomniac	241 (30.7)		Ref.		Ref.	
Insomniac	70 (40.7)		1.59 (1.10-2.8)	0.011	1.43 (1.00-2.03)	0.050
PSQI		<0.001				
Good quality	152 (25.8)		Ref.		Ref.	
Poor quality	159 (43.2)		2.19 (1.66-2.89)	<0.001	1.31 (0.96-1.81)	0.094
BQ		0.001				
Low risk	175 (28.7)		Ref.		Ref.	
High risk	136 (39.1)		1.59 (1.21-2.10)	0.001	1.32 (0.95-1.83)	0.097
**Sleep difficulties**						
Delay in falling asleep		0.064				
Less than once a week	183 (30.3)		Ref.		Ref.	
At least once a week	128 (36.2)		1.30 (0.99-1.72)	0.064	1.18 (0.85-1.63)	0.328
Inability to maintain asleep		<0.001				
Less than once a week	51 (14.1)		Ref.		Ref.	
At least once a week	260 (43.7)		4.73 (3.38-6.63)	<0.001	2.99 (2.05-4.36)	<0.001
Early morning awakenings		<0.001				
Less than once a week	156 (28.0)		Ref.		Ref.	
At least once a week	155 (38.8)		1.63 (1.24-2.14)	<0.001	1.38 (1.00-1.89)	0.050

Notes: ESS = Epworth sleepiness scale; AIS = Athens insomnia scale; PSQI = Pittsburgh sleep quality index; BQ = Berlin questionnaire; cOR = Crude odds ratio; aOR = Adjusted odds ratio; CI = Confidence interval; Model 1 = Crude, unadjusted; Model 2 = Adjusted for socio-demographic characteristics, lifestyle habits, and health related characteristics.

Univariate statistical analysis showed that DL was more frequent in subjects with insomnia (40.7% vs. 30.7%, *p*=0.011), poor sleep quality (43.2% vs. 25.8%, *p*<0.001) and high risk for OSA (39.1% vs. 28.7%, *p*=0.001); no association with excessive daytime sleepiness was found (*p*=0.466).

In multivariate logistic regression analysis controlling for all subjects’ characteristics, the odds of DL remained significantly associated only with insomnia (aOR=1.43, *p*=0.050), while its relation with poor sleep quality (aOR=1.31, *p*=0.094) and the risk for OSA (aOR=1.32, *p*=0.097) were of marginal statistical significance (Table 6). However, in stratifed analysis according to gender, higher odds of DL were significantly associated with insomnia (aOR=1.75, 95% CI=1.05-2.94, *p*=0.030), poor sleep quality (aOR=2.02, 95% CI=1.23-3.33, *p*=0.006) and the risk for OSA (aOR=1.62, 95% CI=0.97-2.72, *p*=0.067) among women but not among men (aOR=1.18, *p*=0.543 for insomnia; aOR=1.39, *p*=0.112 for poor sleep quality; aOR=0.99, *p*=0.960 for the risk for OSA).

Regarding to the basic difficulties of sleep patterns (Table 6), significant increased odds of DL were found among subjects who reported difficulties maintaining sleep (aOR=2.99, *p*<0.001) and early morning awakenings (aOR=1.38, *p*=0.050), but not difficulties initiating sleep (aOR=1.18, *p*=0.328). Again, higher odds for DL were associated with difficulties maintaining sleep (aOR=6.84, 95% CI=3.86-12.11, *p*<0.001), early morning awakenings (aOR=1.70, 95% CI=1.08-2.68, *p*=0.022) and difficulties initiating sleep (aOR=1.54, 95% CI=0.94-2.52, *p*=0.089) among females, although the latter was of marginal significance, but not among males (aOR=0.79, 95% CI=0.50-1.27, *p*=0.331 for difficulties initiating sleep; aOR=1.37, 95% CI=0.77-2.44, *p*=0.291 for difficulties maintaining sleep and aOR=1.10, 95% CI=0.67-1.77, *p*=0.715 for early morning awakenings).

## DISCUSSION

A population based cross-sectional study utilizing self-reported questionnaires was conducted in the primary care setting in northeastern Greece and presented significant associations between short sleep duration and DL among adults irrespective of gender. Moreover, it was revealed that DL prevalence was significantly associated with insomnia, particularly with difficulties maintaining sleep and early morning awakenings.

The high-evidenced prevalence of DL (32.5% of the study population sampled) is consistent with the results of Merekoulias et al. (2014)^32^ who reported a prevalence of 35.7% of DL among individuals over 70 years old with poor sleep quality in Western Greece. Furthermore, DL was more prominent among Greek Muslims affecting 39.2% of our population, compared to Expatriated Greeks and Greek Christians with proportions of 28.8% and 29.9%, respectively. Several studies have also underlined the growing prevalence of DL in minority groups^33,34^. Moreover, rural citizens presented DL twice more frequently when compared to inhabitants of urban areas. This finding probably derives from educational and socioeconomic disparities; hence, people with low educational status from urban areas seem to be more prone to the detrimental effect of DL. Indeed, low financial and educational status in a Korean population favored the development of DL^35,36^. Quite interestingly, the marital status affected the distribution of DL in our sample, with the widowed patients holding the lion’s share, as also proved in the research of Nikparvar et al. (2020)^37^. Additionally, our results demonstrated that factors such as ex-smoking, obesity, sedentary life, and unhealthy diets may contribute to higher DL rates of developing populations, along with previous studies^38,39^. Finally, in keeping with our results emerging evidence from the study of Rekleiti et al. (2012)^40^ demonstrated the independence of DL as a prognostic factor for anxiety.

With regards to sleep duration, our study revealed that short sleepers (<6h) exhibit more than two-fold higher odds for DL compared to normal sleepers (6-8h) (aOR=2.18, *p*<0.001). Furthermore, reduced sleep duration by one hour was associated with a 14% increase in the risk for DL (aOR=1.14, 95% CI=1.00-1.30). Apart from that, sleep duration exhibited significant ability to discriminate subjects with DL (AUC=0.633, 95% CI=0.592-0.675, *p*<0.001) determining the optimal cutoff point of 5:33 hours sleep duration. In contrast, sleeping longer than 8 hours was not associated with DL (aOR=1.02, 95% CI=0.64-1.64).

Available cross-sectional studies have not reached a consensus concerning the relationship between sleep duration and DL. For example, Lin et al. (2017)^10^ and Choi et al. (2008)^11^ utilizing self-reported questionnaires revealed a U-shaped association between sleep duration and low HDL-C among middle-aged and elderly population in Taiwan and sleep duration and low HDL-C and high TG levels among Koreans over the age of 60, respectively. On the other hand, Bjorvatn et al. (2007)^12^ demonstrated that in individuals from the Hordaland County, Norway, TC, and TG levels were higher only among short sleepers. Similarly, Smiley et al. (2019)^41^ concluded that short sleep duration was related to high TG and low HDL-C levels among US citizens that participated in the 2013-14 National Health and Nutrition Examination Survey (NHANES). In contrast, Shin et al. (2016)^13^ exhibited that long, but not short sleep duration was related to low HDL-C levels among a Korean adult population from the Korean National Health and Nutrition Examination Survey. Van Den Berg et al. (2008)^48^ also found that long sleep duration was positively associated with DL investigating a sample of 768 elderly adults from the Netherlands.

Our study did not elicit gender-specific associations between short or long sleep duration and DL. In keeping with our results, Song et al. (2020)^18^ could also not find significant interactions between sleep duration and sex with respect to abnormal serum lipid levels. In contrast, data from the China Health and Nutrition Survey (2009) showed that both short and long sleep duration were associated with higher risks of abnormal serum lipid profiles in women, but not in men^17^. Similarly, Kaneita et al. (2008)^43^ found that both short and long sleep duration were positively associated with high TG and low HDL-C in Japanese females, but not males. Nakanishi et al. (1999)^44^ could also not find a significant association between sleep duration and serum lipid and lipoprotein levels among Japanese men. Finally, Williams et al. (2007)^45^ exhibited that short sleep duration was related to low HDL-C levels in adult American women with type 2 diabetes. Several investigators have tried to explain the gender-specific effects forging the relationship between sleep quantity and DL by implicating bias in self-reporting of sleep^46^ and differences born either by social and household statutes^47^ or by sex hormones variation^48^.

With regards to sleep quality, DL remained significantly associated only with insomnia (aOR=1.43, *p*=0.050) while its relation with poor sleep quality (aOR=1.31, *p*=0.094) and the risk for OSA (aOR=1.32, *p*=0.097) were found of marginal statistical significance. However, in stratifed analysis according to gender, DL was significantly associated with insomnia (aOR=1.75, 95% CI=1.05-2.94, *p*=0.030), poor sleep quality (aOR=2.02, 95% CI=1.23-3.33,*p*=0.006) and the risk for OSA (aOR=1.62, 95% CI=0.97-2.72, *p*=0.067) in females but not males. Concerning insomnia subtypes DL was significantly associated with difficulties maintaining sleep (aOR=2.99, *p*<0.001) and early morning awakenings (aOR=1.38, *p*=0.050), but not difficulties initiating sleep (aOR=1.18, *p*=0.328). Again, DL was associated with difficulties maintaining sleep (aOR=6.84, 95% CI=3.86-12.11, *p*<0.001) and early morning awakenings (aOR=1.70, 95% CI=1.08-2.68, *p*=0.022) solely among females, whereas only females exhibited a marginally significant association between DL and difficulties initiating sleep (aOR=1.54, 95% CI=0.94-2.52, *p*=0.089). Few studies have investigated the relationship between sleep quality and insomnia. Vozoris (2016)^16^ having used data from the 2005-2006 and 2007-2008 National Health and Nutrition Examination Surveys (NHANES) concluded that insomnia symptoms are not associated with DL. Similarly, Zhan et al. (2014) utilizing data from more than 10.000 Chinese individuals, apart from 25% increased odds of elevated TC level only among women experiencing insomnia, did not find significant associations between insomnia and LDL-C, HDL-C, or TG levels among both sexes. In contrast, Rouleau et al. (2017)^49^ exhibited that upon completion of an exercise-based cardiac rehabilitation program, greater improvement in insomnia symptom severity was associated with greater improvements in TC levels. Finally, Chien et al. (2010)^50^ revealed that Taiwanese insomniacs had, surprisingly, lower TC levels compared to non-insomniacs.

Several mechanisms have been implicated in the pathogenetic relationship between inadequate sleep and DL. The study of Corgosinho et al. (2013)^51^ highlighted the burden of short sleep duration on human metabolism, thus inducing increased dietary fat consumption, DL and obesity, which is many times difficult to confront especially among adolescents. For example, sleep restriction strongly infuences metabolic hormones that regulate energy balance, as it has been shown that short sleep duration lowers the blood concentration of leptin that suppresses appetite, and increases the blood concentration of ghrelin, which promotes appetite and sequentially daily dietary intake of cholesterol, trans-fats, and saturated fats^52^. Apart from that, inadequate sleep has been associated with excessive daytime fatigue and consequent reduced engagement in physical activities. Low levels of physical activity have been shown to increase LDL and lower HDL levels^53^. Moreover, it has been presented that short sleep duration by increasing acute stress significantly raises TC and LDL-C levels^54^. In agreement, the evidence of Andersen et al. (2009)^55^ on animal models suggest that the exposure to chronic stressful stimuli resulted in impairment of all parameters of the lipid profile. Finally, it has been proposed that sleep restriction could modify genetic risk factors for adverse blood lipid profiles^56^.

Our analysis manifests several strengths, as it is based on data from a large representative sample of the population in northeastern Greece that provided excellent response rates to sleep quality and quantity and DL measurements (71% compared, for example, to 63% in the Hordaland Health Study)^12^. Moreover, our sampling scheme ensured that the sample was randomly selected and representative of the general population of this area. Limitations of this study lie in the recall bias of self-reported sleep duration as reported sleep duration may be overestimated^57^. Nevertheless, self-report assessments of sleep have been shown to be valid measures compared to quantitative ones based on actigraphy^58^. Apart from that, data were not obtained separately on hypercholesterolemia or hypertrygliceridemia or in patients who were on treatment for dyslipidemia, and therefore the relationship between sleep disturbances with these two dyslipidemic states or patients who were on lipid-lowering agents could not be evaluated. Another limitation involves the properties of the cross-sectional study as causal relationships between sleep pathology and DL could not be determined.

Heterogeneity in sleep duration definition combined with the limited number of relevant studies performed^59,60^ constitute the evidence based statistical interpretation a tedious task^61^. Thus, further prospective studies are required and a longitudinal study on this topic by our research team is scheduled.

## CONCLUSION

To the best of our knowledge, this is the first conducted cross-sectional study in Greece that elucidates an association between short sleep duration, insomnia and DL. Considering its strengths and limitations it could be proposed that early pharmacological, cognitive, and behavioral interventions that improve sleep quality and quantity might serve as preventative measures for DL.
